# Potentially Bioaccessible Phenolic and Antioxidant Potential of Fresh and Stored Lentil Sprouts—Effect of *Lactobacillus plantarum* 299v Enrichment

**DOI:** 10.3390/molecules26082109

**Published:** 2021-04-07

**Authors:** Urszula Gawlik-Dziki, Barbara Baraniak, Małgorzata Sikora, Anna Jakubczyk, Ireneusz Kapusta, Michał Świeca

**Affiliations:** 1 Department of Biochemistry and Food Chemistry, University of Life Sciences, Skromna Str. 8, 20-704 Lublin, Poland; urszula.gawlik@up.lublin.pl (U.G.-D.); barbara.baraniak@up.lublin.pl (B.B.); malgorzata.sikora@up.lublin.pl (M.S.); anna.jakubczyk@up.lublin.pl (A.J.); 2 Department of Food Technology and Human Nutrition, Rzeszów University, 4 Zelwerowicza Street, 35-601 Rzeszów, Poland; ikapusta@univ.rzeszow.pl

**Keywords:** lentil, *Lactobacillus plantarum*, phenolic compounds, antioxidant activity, storage, bioaccessibility

## Abstract

The phenolic and antioxidant potential of potentially bioaccessible fractions of lentil sprouts was studied. Sprouts were cocultivated with a probiotic to obtain a new functional product and further stored in cool conditions. The fraction obtained after buffer extraction and gastric digestion had higher content of phenolics compared to the control (by 20% and 46%, respectively); however, a 9% decrease was observed in samples obtained after gastrointestinal digestion. After gastrointestinal digestion, the highest content of phenolics (278 µg/g d.w.) was determined in the fresh control sprouts. Compounds neutralizing ABTS and hydroxyl radicals, chelating metal ions, and exhibiting strong reducing power were effectively released after gastrointestinal digestion (e.g., the values of the gastrointestinal digestibility index for chelating power and ability to quench hydroxyl radicals significantly exceeded 1 in all studied samples). It was proved that the enrichment of sprouts with a probiotic and further storage significantly improved the antioxidant potential; compared to the fresh control sprouts, an increase by 45% and 10% was determined after the gastric and gastrointestinal digestion, respectively. Lentil sprouts enriched with *L. plantarum* 299v may be a new functional product characterized by the high antioxidant capacity of the potentially bioaccessible fraction.

## 1. Introduction

The so-called health-promoting foods (wholesome foods, super-foods, functional foods) arouse great interest among conscious consumers from developed countries [1]. The social demand for such products results in the introduction of new unconventional technologies and thus, in the development of new products based on commonly known components with documented prohealth properties. Fortification is a common way to obtain such products [2,3]. This method is usually used to reduce or eliminate micronutrient deficiencies, improve nutritional quality via vitamin and mineral supplementation, and promote dietary diversification and home food production [1].

In recent years, agronomic intervention and genetic selection have contributed to an increase in the concentrations of desirable components in edible plants [3]. However, techniques that do not require genetic modification arouse greater social acceptance. One of these methods with documented effectiveness and security is elicitation [4]. The enrichment of plant foods with probiotic bacteria is a variation of this technique [5].

Probiotic bacteria have become increasingly popular during the last two decades due to their beneficial influence on human health; thus, the food industry has been very active in studying and promoting these probiotic agents [6]. Within this market, probiotics have been incorporated in various products. Currently, the issue of fruits and vegetables containing probiotic strains is gaining considerable consumers’ interest [5,7]. The effectiveness of *Lb. plantarum* 299v in the treatment of gastrointestinal diseases, allergic diseases, obesity, insulin resistance syndrome, type 2 diabetes, and nonalcoholic fatty liver disease has been confirmed [8,9,10]. One of the newest products dedicated for vegans and/or the increasing number of consumers suffering from lactose intolerance or allergies are legume sprouts enriched with *Lactobacillus plantarum* 299v [9]. In these sprouts, the lactic acid bacterial (LAB) count was 7.61 log10 CFU/g f.m. and increased during storage in cold conditions. Moreover, in probiotic-rich sprouts, LAB accounted for 98% of the total microorganisms.

Lentils are consumed as a whole food in more than 100 countries. This legume has an excellent nutrient profile and favorable levels of antioxidants; however, antinutritive factors such as condensed tannins and phytic acid reduce their nutritive value [10]. During germination, the content of low-molecular-weight phenolic compounds does not vary appreciably; moreover, some conditions increase the phenolic contents in sprouts [11]. It is also well documented that abiotic stresses (e.g., hydrogen peroxide, salinity), components of the cell wall (chitosan, peptidoglycans), and microbial metabolites may increase the de novo synthesis of phenolic compounds by induction of the phenylpropanoid pathway [12,13,14,15,16].

Phenolic compounds have multiple additional physiological roles in plants and have also been associated with the flavor and color characteristics of plant foods [17,18]. Moreover, in recent years, phenolic compounds have been intensively investigated to determine their potential health-promoting effects, and their best-studied biological activity is based on their antioxidant capacity [10,19]. The biological properties of antioxidants strongly depend on their bioaccessibility. Bioaccessibility may be defined as the amount of any food component that is released from the food matrix, is detectable in the small intestine, and may be able to pass through the intestinal barrier [20]. Potential bioaccessibility is usually evaluated by in vitro digestion procedures, generally simulating gastric and small intestinal digestion. Therefore, in vitro digestion models are widely used for studying structural changes, digestibility, and the release of food components [19,20,21]. Generally, despite the low pH in the stomach, low-molecular-weight polyphenols are stable, and only glucose-bound polyphenols are partially hydrolyzed. At this step, some phenolics (e.g., anthocyanins) can be absorbed via active transport [22]. Monomeric polyphenols, e.g., flavonoids, are usually catabolized to chain fission products by intestinal bacteria in the colon [20,23]. It has also been proven that probiotic strains (e.g., *Saccharomyces cerevisiae* or *L. plantarum*) may represent an intermediary for increasing the antioxidant bioactivity (bioaccessibility); thus, new food products enriched with these functional organisms are developed [24]. On the other hand, the physiological functionality of bacterial strains can be enhanced in the presence of polyphenols, e.g., the interaction of quercetin with *L. fermentum* and *L. plantarum* strains [25]. Although in vitro models used for bioavailability studies have some limitations (ignored absorption, phase I and II reactions, distribution to the target organs), they provide some information about the liberation, solubilization, and interaction of bioactive compounds. It has been proven that the rate of liberation from the food matrix and transformation during transition through the upper parts of the digestive tract significantly affects the bioaccessibility and further metabolism of phenolics. Additionally, analyses of digests may be useful for estimation of “local” activity, e.g., against inflammation of gastric mucosa or stomach cancers [26].

Therefore, the aim of our work was to assess the impact of probiotic bacteria on changes in the polyphenol profile and the antioxidant capacity of potentially bioavailable compounds contained in lentil sprouts, as well as their storage stability.

## 2. Materials and Methods

### 2.1. Materials

All chemicals used for the cultivation of sprouts and microbiological media were purchased from the Sigma—Aldrich company (Poznan, Poland) and BTL Ltd. (Łodz, Poland). Lentil seeds were purchased from the PNOS S.A. in Ozarów Mazowiecki, Poland. The strain of *Lactobacillus plantarum* 299v was isolated from a commercial probiotic preparation. The culture was tested for cell morphology. Biochemical tests and 16S rRNA sequencing were performed as well.

### 2.2. Sprouting Conditions 

Sprouting was carried out at 25 °C as described previously [9]. Briefly, seeds were soaked in distilled water (C, control) or a *Lb. plantarum* 299V water suspension (LP, 1 × 10^8^ CFU per 1 g of seeds) for 4 h. The seeds were dark-germinated in a growth chamber (SANYO MLR350H) on Petri dishes (Ø 125 mm) lined with absorbent paper. 4-day-old sprouts were manually collected (fresh sprouts) or stored in polypropylene boxes at 4 °C for 7 days (stored sprouts). After freeze-drying, the sprouts were milled, sieved (0.5 mm), and stored in polypropylene boxes at −60 °C.

### 2.3. Phenolic Content and Antioxidant Activity

#### 2.3.1. Extraction Procedure

##### Buffer Extractable Fractions

The powdered samples of sprouts (500 mg, particle size < 0.2 mm) were mixed with 5 mL of saline buffer (PBS, pH 7.4). Then, the samples were sonicated (3 intervals of 30 s, at room temperature (25 ±  1 °C)). The ultrasonic treatment was performed using an ultrasonic cleaner (42 kHz, 135 W; Branson Ultrasonic Corporation, Brookfield, WI, USA). After that, the samples were centrifuged (15 min 6900× *g*) and the supernatants were kept at −20 °C before analysis as buffer extracts (BE).

##### In Vitro Digestion

In vitro digestion was performed as described previously [21]. For analysis of the fraction after gastric digestion, the action of enzymes was stopped by adding pure methanol (1:1 ratio) (extracts after gastric digestion (GI)). For analysis of the potentially bioaccessible fraction, samples, after gastrointestinal digestion, were mixed with an equal volume of methanol to stop the digestion. After centrifugation (15 min 6900× *g*), the supernatants were used for the study (extracts after gastrointestinal digestion (GDI)).

#### 2.3.2. Phenolic Assay

The crude extract was suspended in water (10 mL) and passed through a C18 Sep-Pak (360 mg, 55–105 μm) cartridge (Waters Associates, Milford, MA, USA) preconditioned with water. The cartridge was washed first with water (10 mL) to remove sugars and then with MeOH (10 mL) to elute phenolics. This fraction was evaporated to dryness and redissolved in 50% MeOH for analyses. 

Structural information and general phenolic profiles were gathered using a Waters Aquity UPLC system consisting of a binary solvent manager, sample manager, PDA detector, and triple quadrupole detector (TQD) operating in the negative electrospray mode. The ion source parameters were as follows: cone voltage 35 V, capillary voltage 3 kV, extractor 3 V, RF lens 100 mV, source temperature 120 °C, desolvation temperature 350 °C, desolvation gas flow 800 L/h, cone gas flow 100 L/h, and collision gas flow 300 μL/min. The collision cell parameters were as follows: entrance −2, exit 0.5, and collision energy 22 eV. The parameters of quadrupole 1 were set to achieve maximal mass resolution: both LM and HM resolutions were set to 15 and ion energy was set to 0.8. The collision cell parameters for the MS/MS experiments were as follows: gas collision pressure (argon), 1.5.10-3 mbar, and collision energy 15 or 30 eV. Acquisition in both MS scan and product ion scan modes was performed in the centroid mode monitoring from 100 to 1200 *m*/*z* value. Phenolic acids were separated on a 100 mm × 2.1 mm i.d., 1.7 μm Acquity BEH column (Waters), using the linear 8.5 min gradient from 80 to 100% of solvent B (40% acetonitrile containing 0.1% formic acid) in solvent A (water containing 0.1% formic acid) with a flow of 0.35 mL min^−1^. Results were expressed in µg per g of dry weight (d.w.) as equivalents of kaempferol 3-O-glucoside [27].

#### 2.3.3. Antioxidant Activity

##### Ability to Quench ABTS Radicals

The experiments were carried out using the ABTS decolorization assay [28]. The free radical scavenging ability was expressed as Trolox (TE, 6-hydroxy-2,5,7,8-tetramethylchroman-2-carboxylic acid) equivalents in mg per g of dry weight (d.w.).

##### Ability to Quench Hydroxyl (OH^•^) Radicals

The OH^•^ scavenging ability was assessed according to Su, Wang, & Liu [29]. Hydroxyl radicals were generated via Fenton reaction in the system of FeSO_4_ and H_2_O_2_.

The free radical scavenging ability was expressed as Trolox (TE, 6-hydroxy-2,5,7,8-tetramethylchroman-2-carboxylic acid) equivalents in mg per g of d.w.

##### Ferric Reducing Power (RP)

Reducing power was determined as in Oyaizu [30]. It was expressed as Trolox (TE, 6-hydroxy-2,5,7,8-tetramethylchroman-2-carboxylic acid) equivalents in mg per g of dry weight (d.w.).

##### Metal Chelating Activity (CHP)

Chelating power was determined with the method proposed by Guo, Lee, Chiang, Lin, & Chang [31]. It was expressed as EDTA equivalents (EDTA, ethylenediaminetetraacetic acid) in mg per g of d.w.

##### Total Antioxidant Index

Four complementary antioxidant methods were integrated to obtain the total antioxidant activity index (*TAI*). The index may be useful for the evaluation of total antioxidant potential in respect to the fresh control. The *TAI* was calculated as the sum of relative activities (*RA*) for each antioxidant method divided by the number of methods.

(1) $$TAI=\frac{\Sigma^{}RA_{\left(n\right)}}{4}$$

*RA* was calculated as follows:

(2) $$RA*=\frac{A_{x}}{A_{c}}$$

where: *Ac*-activity of the studied sample for the method, *Ac*-activity of the fresh control sprouts determined for the method.

#### 2.3.4. Theoretical Approaches

The following factors were determined to better understand the potential bioaccessibility of biologically active compounds [32]:

(1) the gastric digestibility index (GA), which is an indicator of the bioaccessibility of gastrically released antioxidants:
  GA = A_GD_/A_BE_ (3)
(2) the gastrointestinal digestibility index (IA), which is an indicator of the bioaccessibility of gastrointestinally released antioxidants:
  IA= A_GDI_/A_BE_ (4)
(3) the relative intestinal digestibility index (RIA), which is an indicator of the susceptibility of antioxidants to intestinal digestion:
  RIA = A_GDI_/A_GD_ (5)
  where: A_BE_ is the activity of buffer extract (BE), A_GD_ is the activity of the extracts after simulated gastric digestion (GD), and A_GDI_ is the activity of the extracts after simulated gastrointestinal digestion (GDI).

#### 2.3.5. Statistical Analysis

All experimental results are means ± S.D. of three parallel experiments. One-way analysis of variance (ANOVA) and Tukey’s post hoc test were used to compare the groups. Differences were considered significant at *p* ≤ 0.05.

## 3. Results and Discussion

### 3.1. Phenolic Content

Lentil sprouts were found to be a good source of phenolic compounds, especially flavonoid derivatives (Table 1). The structure of the compounds was determined based on the previous studies by Zuchowski, Pecio, and Stochmal [33], where they were isolated and their structure was fully confirmed by NMR experiments. The main flavonoid derivative identified in the BE extracts from the control sprouts (C) and the probiotic-enriched sprouts (LP) were kaempferol 3-O-β-glucopyranosyl(1→2)-α-rhamnopyranosyl-7-O-α-glucopyranoside (C3), kaempferol 3-O-[β-glucopyranosyl(1→2){α-rhamnopyranosyl (1→6)}-β-galactopyranoside]- -7-O-α-rhamnopyranoside (C2), and catechin gallate (C1) (Table 2). Importantly, their content was higher in the probiotic-rich sprouts; however, the differences were not statistically significant in the case of C2 and C3. Statistically significant differences were observed in the case of C4, C5, C8, C9, C10, C11, and C12; their content in the LP sprouts was about 2-fold higher than in the control sprouts. As reported by Troszynska et al., the most abundant phenolics detected in lentils are compounds with a flavanol structure (i.e., monomers, oligomers, and gallate derivatives), with catechin glucoside as the predominant flavanol [17]. There are some qualitative differences observed between the studies; however, they may be caused by the use of different extraction systems for the separation of phenolic fractions and the type of tested samples (raw seeds vs. sprouts). Derivatives of quercetin and kaempferol were detected as well. Additionally, kaempferol and their derivatives: kaempferol dihexoside, kaempferol 3-O-rutinoside, quercetin 3-O-glucoside, luteolin rhamnose hexose, kaempferol rhamnose-dihexoside, and kaempferol 3-O-glucoside were identified in the fiber fractions of germinated lentils [34,35].

The digestion process usually affects bioactive compounds; thus, it is imperative to determine whether it is reflected in their beneficial effects, including antioxidant activity. It is widely known that gastrointestinal processing results in substantial changes in the phenolic profile of food [22]. This is also clearly visible in our study. After the gastric digestion of the control sprouts, a significant decrease in the content of all previously identified compounds was found. Most importantly, no C4, C5, and C6 were found in the samples after the simulated digestion. However, quercetin 3-O-[(6-O-E-caffeoyl)-β-glucopyranosyl (1→2)]-β-galactopyranoside-7-O- (2-O-E-coumaroyl)-β- glucuropyranoside (C13) was identified after gastric digestion. It is generally difficult to determine the main pattern, as the differences are not statistically significant in most of the identified compounds. However, by comparing the sum of the compounds, it can be declared that the addition of LP resulted in their slight increase. The further digestion process resulted in slight changes in the phenolic profile of the analyzed samples. The individual differences probably resulted from the pH conditions rather than from the action of digestive enzymes (Table 2). 

Most phenolic compounds remain stable during salivary and gastric digestion, probably due to the protective action of acid pH during the gastric step [36]. Gayoso et al. reported a significant decrease in the amount of rutin, caffeic acid, and rosmarinic acid after gastrointestinal digestion, recovering only 37%, 8%, and 27% of the initial concentration, respectively [37]. Losses of phenolic compounds from chokeberry and red wine after gastrointestinal digestion were confirmed as well [25,26]. These compounds are highly sensitive to the mild alkaline conditions present in the small intestine, where most dietary polyphenols are degraded or transformed into other compounds [38,39].

Nevertheless, high stability after in vitro pancreatic digestion of pure quercetin and catechin [40] as well as ellagic acid [41] has been reported. In general, the differences among studies may result from the effect of the food matrix and the different experimental conditions applied [37]. Some losses of phenolics (compared to buffer extracts) may be due to their interaction with digestive enzymes [42]. Such behavior was previously observed for soybean isoflavones [43] or almond phenolics [44]. In vitro models have some limitations, but it has been proven that they may provide interesting data about the behavior of phenolic antioxidants in the upper parts of the digestive tract.

Sprouts are usually stored before consumption; thus, all analyses involved the fresh sprouts and those stored in cooling conditions. Regardless of the sample (probiotic enrichment, stage of digestion), the total phenolic content in the cold-stored sprouts was generally lower than in the fresh sprouts. Taking into account only the stored sprouts, some significant differences in the individual compounds were found between the control and probiotic-rich samples. The LP sprouts were a richer source (compared to the control sprouts) of buffer-extractable phenolics (C2, C4, C8, C11, and C12). Interestingly, the content of C11 and C12 was significantly higher in the stored than in the fresh LB sprouts (Table 2 and Table 3). As in the case of the fresh sprouts, the simulated digestion strongly influenced the phenolic profile of all samples. Analogically to the fresh sprouts, no C4, C5, and C6 compounds were found after gastric digestion, but C13 was identified; however, the content was significantly lower than in the fresh sprouts. Most importantly, gastric digestion of the stored LP sprouts released significantly higher amounts of phenolics compared to the control stored sprouts; their level was comparable to that in the fresh LP sprouts. Taking into account the individual compounds, the enrichment with LP resulted in an increase in C2, C3, C9, C10, C11, and C12, which was not observed in the fresh sprouts. The further digestion resulted in slight changes in the phenolic profile of the LP samples, whereas an interesting trend was observed in the case of the stored control sprouts. In this case, higher phenolic content was detected after gastrointestinal digestion (compared to the gastric digestion) (Table 3).

Lentil sprouts enriched with LP are a new functional product proposed for the first time [9]; hence, there are no reports in the current literature showing changes in the phenolic profile induced by colonization with probiotic bacteria. However, there have been many studies on the effect of biotic elicitors based on microorganisms and fungi employed in the overproduction of phenolics in plants [45]. Złotek & Świeca found that the application of yeast extracts caused an increase in the total phenolic compounds that was positively correlated with the antioxidant and anti-inflammatory activity of lettuce leaves [14]. As shown by Swieca et al., coculturing of lentil and adzuki bean sprouts with probiotic yeasts improved the microbiological quality of the sprouts and increased the antioxidant capacity of the potentially bioaccessible fraction [46]. Similarly, Portu, López, Baroja, Santamaría, & Garde-Cerdán showed that foliar treatments with yeast extracts increased the anthocyanin and stilbene content in grapes and wine, compared to the control [47]. Biotic elicitors, i.e., extracts from *Saccharomyces cerevisiae* (SC), were also used by Gawlik-Dziki et al. to modify the nutraceutical potential of sprouted wheat [48]. The elicitation slightly decreased the content of protocatechuic, *p*-hydroxybenzoic, vanillic, and syryngic acids, whereas treatment with SC caused an increase in the content of caffeic and *t*-synapinic acids.

In the current literature, there are no detailed studies on the profile of the phenolic compounds of stored lentil sprouts. In our previous studies, no significant effect from storage on the total phenolic and flavonoid content in lentil sprouts was observed [49]. However, a decrease in their content was found in both the control and LP-enriched sprouts in the present study, probably due to the greater precision of the methodology used in this research (Folin—Ciocalteu reagent vs. UPLC- MS/MS method). In summary, it can be concluded that the enrichment with the probiotic did not change the phenolic profile but influenced the level of individual compounds. The enrichment with LP caused an increase in the total phenolic content in comparison to the control sprouts, irrespective of the type of the extract studied. The LP sprouts proved to be a good source of potentially bioavailable phenolics with stability during storage. The stored LP sprouts were more susceptible to gastric digestion than the control sprouts. 

### 3.2. Antioxidant Assays

Different antioxidants characterized by different modes of action can be found in a wide range of concentrations in vegetal tissues; therefore, different procedures should be used to quantify the antioxidant capacity of the material. There are several assays, such as the ferric reducing antioxidant power (FRAP) and 2,2-diphenyl-1-picrylhydrazyl (DPPH) or 2,2′-azinobis-(3-ethylbenzothiazoline-6-sulfonic acid) (ABTS), which are simple, cost-ffective, and easy to interpret [50,51,52,53].

As presented in Table 4, the lentil sprouts appeared to be a good source of buffer-extractable and potentially bioaccessible antioxidants with multidirectional activity. Especially high TAEC values were obtained for reducing power (RP), which is considered by many researchers as a determinant of total capacity [36,37]. The LP enrichment of the lentil sprouts resulted in an increase in this parameter, irrespective of the type of material and extract. For example, an increase by about 18% for BPS, 21% for extracts after gastric digestion, and 24% for extracts after gastrointestinal digestion was detected in the fresh sprouts. This tendency is especially clearly visible in the case of compounds released after the first stage of the simulated digestion (41%) (Table 4).

The ability to quench free radicals is one of the most frequently determined and compared activities. In our study, two methods were used: activity against synthetic ABTS free radicals, and against hydroxyl radicals, naturally occurring in the human organism. The enrichment of the lentil sprouts with the probiotic significantly increased the antiradical activity (ABTS test) in the case of buffer-extractable compounds and those released after gastric digestion, whereas a significant decrease in this activity was observed after the second stage of digestion. A similar relationship was found for the stored sprouts (Table 4). Taking into account the ability to neutralize OH radicals, a negative effect of the enrichment was found in the case of the buffer extracts from the fresh sprouts (a decrease by about 36%). After step I and II of digestion, a slight increase (by approximately 3%) was observed, but a slight decrease in this activity was determined after the storage in the case of the buffer extracts and those obtained after gastric digestion (2% and 7%, respectively). A slight enhancement (6.47%) of antiradical activity was observed in samples obtained after the second stage of digestion. In the case of chelating power, a positive effect of the probiotic enrichment was observed in the fresh control sprouts only in the PBS extract (an increase by about 19%). In the stored sprouts, a negative effect of the enrichment was found in the case of the buffer-extractable fraction and that obtained after gastric digestion (about 16% and 6%, respectively). Summarizing, compounds neutralizing free ABTS radicals were released after gastric digestion, whereas compounds with the ability to chelate metal ions and neutralize OH radicals, and characterized by strong reducing power, were more effectively released after gastrointestinal digestion. This indicates extractor-like action in the gastrointestinal tract and suggests a potential role of nonphenolic compounds (for example, peptides) released during digestion in determining the total antioxidant activity of food [38,39].

An important issue in the case of less processed food is its storage quality. For this reason, the effect of storage of the control and enriched sprouts on their antioxidant activity was determined. In general, storage positively influenced the reducing power of the buffer-extractable fractions and those released after gastric digestion, from the control and enriched sprouts, while a slight decrease in this activity was observed for the fraction obtained after gastrointestinal digestion (4.37% for the control and 11.97% for the LP sprouts, respectively) (Table 4). Positive effects of cold storage were also observed in the case of the chelating activity of the potentially bioaccessible fraction obtained from the control sprouts (an increase by about 12%) and fractions released after step I and II of digestion of the enriched sprouts. Interesting results were obtained for antiradical activities. The analysis of the ability to scavenge free ABTS radicals revealed a negative effect of storage in the case of the control sprouts (all kinds of extracts). Importantly, the storage of the enriched sprouts resulted in an increase in this activity, especially in the PBS-extractable fraction (an increase by about 17%). Particularly significant storage effects were found in the case of the ability to neutralize free hydroxyl radicals. A significant increase in this activity was observed in all of the samples, whereas a higher rise was determined for the PBS extracts from the control and LP sprouts (about 2.8- and 4.8 fold, respectively). In our previous study, the reducing potential of the potentially bioaccessible fraction of stored lentil sprouts was elevated by 40%, 31%, and 23% in 3-, 4-, and 5-day-old sprouts stored for 1 week at 4 °C, respectively [49].

**Figure 1:**
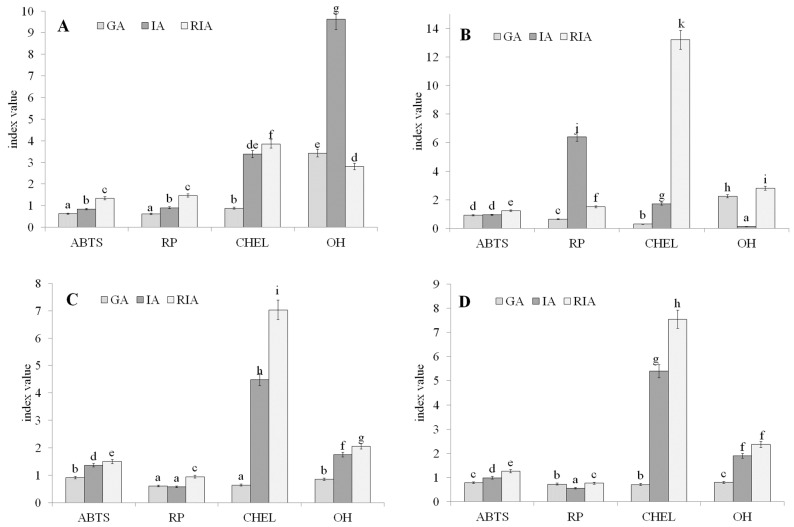
Comparison of the gastric digestibility index (GA), the gastrointestinal digestibility index (IA), and the relative intestinal digestibility index (RIA): (**A**) fresh control sprouts; (**B**) fresh probiotic-rich spouts; (**C**) stored control sprouts, (**D**) stored probiotic-rich sprouts. ABTS—ability to quench ABTS radicals; RP—reducing power; CHP—chelating power; OH—ability to quench OH radicals. Means (±SD) in columns followed by different letters are significantly different (*n* = 9; *p* ≤ 0.05).

The key issue in the case of functional foods is the bioavailability of bioactive compounds. Various models of the human gastrointestinal tract, very often used in model systems, allow researchers to partially illustrate the influence of conditions occurring during digestion on changes in antioxidant activity. This study compares the potential bioaccessibility after two steps of in vitro digestion, the gastric (GD) and gastrointestinal (GDI) stages. In the case of the fresh control sprouts, the highest bioaccessibility was determined for compounds with an ability to scavenge ABTS free radicals and chelate transition metal ions, as well as the reducible ones released after the second digestion step (GDI), while digestion in the simulated stomach released compounds neutralizing OH radicals (Figure 1A). The changes in this activity may be caused by the hydrolysis of inactive complexes between phenolics and food matrix components (especially proteins) and/or the multidirectional activity of peptides released during simulated digestion, as well as pH changes, among which the key role is played by acidification and realkalization. The preservation of phenolics has a great impact on the quality of plant foods due to the involvement of phenols not only in enzymatic browning reactions but also in antioxidant capacity.

Kevers et al. found that, in general, phenolic content increased during storage [54]. Increased levels of antioxidant capacity generally accompanied this increase. An increase in the antioxidant capacity was observed in yellow pepper, broccoli, plum, citrus, and garlic. As shown by Goyal, Siddiqui, Upadhyay, & Soni, during storage of mung beans at room and low temperature, the levels of ascorbic acid, total phenols, and antioxidant activity in sprouts first increased and then decreased significantly [55]. In a study conducted by Yamdeu Galani et al., all phenolic acids in potato, except *p*-coumaric acid, as well as antioxidant activity against both DPPH and ABTS radicals, increased with storage [56].

The research hypothesis assumed that the enrichment of lentil sprouts with LP would result in changes in their multidirectional antioxidant activity. Interestingly, the addition of the probiotic did not affect the bioaccessibility of antiradicals (active against ABTS), while significant differences in other activities were found. The presence of LP increased the potential bioaccessibility of reductive compounds after stage II of in vitro digestion. Importantly, a negative influence on the ability to quench OH radicals and chelating power was observed (Figure 1B). Interestingly, in spite of the decrease in the potential bioaccessibility of chelating compounds (compared to the control sprouts), the strong influence of intestinal digestion on this activity was confirmed (RIA value 13.21) (Figure 1B). Storage resulted in a slight but statistically significant increase in the bioaccessibility of compounds with antiradical activity (against ABTS) and those able to chelate metal ions released after the second stage of the digestion. A decrease in bioaccessibility was found in the case of compounds with reducing potential and those quenching OH radicals (in the case of the control sprouts) (Figure 1C). Similar relationships were confirmed for the LP sprouts (vs. the fresh ones) in the case of the ability to neutralize ABTS radicals and reducing power, while an increase in potential bioaccessibility was observed in the case of compounds with the ability to chelate metal ions and OH radical scavengers (Figure 1D). These phenomena may be partly explained by the fact that the number of bacteria increased during the storage of the enriched sprouts [9], and their metabolism may have caused the changes in the profile of phenolic compounds, antioxidant activity, and potential bioaccessibility of antioxidative compounds. Growing bacteria utilized nutrients and water from sprouts, simultaneously releasing bioactive compounds from inactive complexes and increasing their availability.

Lentil sprouts enriched with *L. plantarum* 299v may be a new functional product proposed for the first time. In summary, probiotic enrichment of the lentil sprouts did not change the phenolic profile but influenced the levels of the individual compounds. The probiotic-rich sprouts were found to be a good source of potentially bioavailable phenolics, especially flavonoid derivatives, exhibiting stability during storage. The total phenolic content in the cold-stored sprouts was generally lower than in the fresh sprouts; however, there were significant differences in the individual compounds. The sprouts were assessed as a good source of buffer-extractable and potentially bioaccessible antioxidants with multidirectional activity. Compounds characterized by the ability to neutralize free ABTS radicals were released after gastric digestion, whereas compounds able to chelate metal ions, neutralize OH radicals, and exhibit strong reducing power were more effectively released after gastrointestinal digestion.

## Figures and Tables

**Table 1:** Individual phenolic compounds identified by UPLC-PDA-MS/MS in lentils.

Abbreviation	Compound	Rt	[M-H]^−^ *m*/*z*	
		min	MS	MS/MS
C1	Catechin gallate	3.08	441	289
C2	Kaempferol3-*O*-[β-glucopyranosyl(1→2){α-rhamnopyranosyl(1→6)}-β-galactopyranoside]-7-*O*-α-rhamnopyranoside	3.51	901	755, 285
C3	Kaempferol 3-*O*-β-glucopyranosyl(1→2)-α-rhamnopyranosyl-7-*O*-α-glucopyranoside	3.55	755	593, 285
C4	Kaempferol 3-*O*-[(6-*O*-E-*p*-coumaroyl)-β-glucopyranosyl(1→2)]-β-galactopyranoside-7-*O*- β-glucuropyranoside	4.18	931	755, 609, 285
C5	Unidentified kaempferol derivative	4.23	655	563, 285
C6	Kaempferol 3-*O*-β-glucopyranosyl(1→2)-α-galactopyranoside-7-*O*-α-glucuropyranoside	4.28	785	609, 285
C7	Kaempferol 3-*O*-[(6-*O*-E-feruolyl)-β-glucopyranosyl(1→2)]-β-galactopyranoside-7-*O*- β-glucuropyranoside	4.44	961	785, 609, 285
C8	Kaempferol 3-*O*-{[(6-*O*-E-*p*-coumaroyl)-β-glucopyranosyl(1→2)]-α-rhamnopyranosyl(1→6)}-β-galactopyranoside]-7-*O*-α-rhamnopyranoside	4.6	1047	901, 755, 285
C9	Quercetin 3-*O*-[(6-*O*-E-caffeoyl)-β-glucopyranosyl(1→2)]-β-galactopyranoside-7-*O*- β-glucuropyranoside	4.79	963	787, 625, 301
C10	Kaempferol 3-*O*-[(6-*O*-E-caffeoyl)-β-glucopyranosyl(1→2)]-β-galactopyranoside-7-*O*- β-glucuropyranoside	4.83	947	771, 609, 285
C11	Quercetin 3-*O*-[(6-*O*-E-*p*-coumaroyl)-β-glucopyranosyl(1→2)]-β-galactopyranoside-7-*O*- β-glucuropyranoside	4.86	947	771, 625, 301
C12	Quercetin 3-*O*-[(6-*O*-E-feruloyl)-β-glucopyranosyl(1→2)] -β-galactopyranoside-7-*O*- β-glucuropyranoside	4.91	977	801, 625, 301
C13	Quercetin 3-*O*-[(6-*O*-E-caffeoyl)-β-glucopyranosyl(1→2)]-β-galactopyranoside-7-*O*- (2-*O*-E-caffeoyl’)-β-glucuropyranoside	5.1	1125	963, 787, 625, 301

**Table 2:** Qualitative and quantitative analysis of phenolics from the fresh control and probiotic-rich sprouts.

Compound [µg/ g d.m.]	BE		GI		GDI	
	C	LP	C	LP	C	LP
C1	162 ± 8.0 c	201 ± 6.6 d	4.00 ± 0.35 b	2.4 ± 0.54 a	2.3 ± 0.23 a	1.9 ± 0.81 a
C2	19 8 ± 4.6 d	211 ± 11 d	142 ± 1.9 b	158 ± 3.1 c	143 ± 0.23 b	115 ± 8.9 a
C3	341 ±29 b	371 ± 19 b	84 ± 9.3 a	90 ± 10 a	89 ± 21 a	95 ± 0.66 a
C4	6.9 ± 2.05 a	12 ± 0.8 b	tr.	tr.	tr.	tr.
C5	12 ± 2.9 a	25 ± 2.5 b	tr.	tr.	tr.	tr.
C6	7.7 ± 1.2 a	7.8 ± 0.55 a	tr.	tr.	tr.	tr.
C7	10 ± 2.6 c	14 ± 1.2 d	3.3 ± 0.13 a	3.6 ± 0.02 a	6.3 ± 2.7 b	5.1 ± 2.06 ab
C8	8.8 ± 2.3 d	14 ± 1.1 e	6.0 ± 0.70 c	5.9 ± 1.7 c	3.5 ± 0.5 b	2.8 ± 2.05 a
C9	6.5 ± 2.08 ab	14 ± 0.6 c	6.2 ± 0.32 b	4.9 ± 0.56 a	5.9 ± 2.8 ab	6.6 ± 5.6 ab
C10	8.3 ± 2.8 a	25 ± 2.1 b	7.9 ± 1.3 a	7.2 ± 1.5 a	7.7 ± 3.5 a	6.4 ± 4.9 a
C11	12 ± 2.8 a	31.5 ± 1.8 b	10.4 ± 1.5 a	8.5 ± 0.57 a	12.10 ± 4.40 a	9.9 ± 6.3 a
C12	10.1 ± 2.4 b	23 ± 2.0 c	7.9 ± 1.67 b	8.8 ± 0.16 ab	7.9 ± 2.9 ab	6.6 ± 2.2 a
C13	tr.	tr.	4.7 ± 1.5 c	4.4 ± 2.8 c	0.65 ± 0.19 ab	1.6 ± 0.21
Sum	787 ± 16 b	951 ± 13 c	277 ± 5.2 a	293 ± 19 a	278 ± 4.9 a	250 ± 34 a

Means (±SD) in columns followed by different letters are significantly different (*n* = 9; *p* ≤ 0.05). C—control sprouts, LP—sprouts enriched with the probiotic, BE—buffer extract, GD—extract after gastric digestion, GDI—extract after gastrointestinal digestion.

**Table 3:** Qualitative and quantitative analysis of phenolics from the stored control and probiotic-rich sprouts.

Compound [µg/ g d.m.]	BE		GI		GDI	
	C	LP	C	LP	C	LP
C1	198 ± 3.58 c	201 ± 15.0 c	3.61 ± 0.65 ab	4.29 ± 0.32 b	4.14 ± 0.95 ab	3.27 ± 0.21 a
C2	141 ± 3.5 e	187 ± 2.7 f	93.6 ± 0.47 a	130 ± 1.27 d	123 ± 3.7 c	107 ± 2.1 b
C3	263 ± 16 c	266 ± 46 c	65.7 ± 2.90 a	74.9 ± 0.35 b	72.6 ± 1.3 b	64.9 ± 4.7 a
C4	5.12 ± 0.56 a	7.57 ± 0.9 b	tr.	tr.	tr.	tr.
C5	13.6 ± 0.51 a	11.9 ± 4.87 a	tr.	tr.	tr.	tr.
C6	8.41 ± 2.69 a	11.1 ± 2.39 a	tr.	tr.	tr.	tr.
C7	8.63 ± 3.55 bcd	13.8 ± 2.53 d	3.08 ± 0.62 a	4.45 ± 0.59 b	5.27 ± 0.12 b	5.70 ± 1.99 abc
C8	5.46 ± 4.03 abc	13.7 ± 2.52 d	4.34 ± 0.22 b	6.68 ± 0.87 c	5.78 ± 3.45 abc	2.95 ± 0.98 a
C9	9.09 ± 5.61 abc	14.0 ± 5.30 bc	6.57 ± 1.31 a	10.4 ± 2.1 bc	11.7 ± 2.51 bc	8.18 ± 3.20 ab
C10	14.2 ± 9.5 abcd	27.9 ± 9.51 cd	8.73 ± 2.46 a	19.3 ± 3.1 cd	12.0 ± 1.49 ab	15.1 ± 3.44 bc
C11	18.3 ± 8.70 abc	49.5 ± 12.3 d	11.6 ± 2.77 a	24.1 ± 3.66 c	16.5 ± 0.91 b	18.0 ± 8.2 abc
C12	18.4 ± 8.45 bc	36.4 ± 10.6 d	6.88 ± 1.35 a	15.5 ± 2.78 c	10.5 ± 0.32 b	12.9 ± 3.98 bc
C13	tr.	tr.	0.70 ± 0.30 a	1.95 ± 0.35 b	0.95 ± 0.09 a	1.28 ± 0.23 ab
Sum	706 ± 24 e	844 ± 28 f	204 ± 6.0 a	292 ± 12 d	263 ± 1.1 c	239 ± 15 b

Means (±SD) in columns followed by different letters are significantly different (*n* = 9; *p* ≤ 0.05). C—control sprouts, LP—sprouts enriched with the probiotic, BE—buffer extract, GD—extract after gastric digestion, GDI—extract after gastrointestinal digestion.

**Table 4:** Antioxidant capacity of phenolics from the stored control and probiotic-rich sprouts.

	Sprouts		ABTS [mg TE/ g d.w.]	RP [mg TE/ g d.w.]	CHP [mg EDTA/ g d.w.]	OH [mg TE/ g d.w.]	TAI
BE	C	F	0.94 ± 0.080 a	5.45 ± 0.11 a	1.70 ± 0.92 a	0.12 ± 0.0002 a	1.00
	C	S	0.90 ± 0.073 b	8.17 ± 0.08 b	1.79 ± 0.15 b	0.46 ± 0.004 b	1.24
	LP	F	1.49 ± 0.057 c	6.41 ± 0.038 c	2.12 ± 0.17 c	0.077 ± 0.0106 c	1.00
	LP	S	1.11 ± 0.072 d	9.54 ± 0.025 e	1.51 ± 0.31 d	0.45 ± 0.004 b	1.38
GD	C	F	0.86 ± 0.031 f	3.33 ± 0.12 f	0.51 ± 0.14 e	0.27 ± 0.013 d	1.00
	C	S	0.82 ± 0.028 g	4.95 ± 0.10 g	1.14 ± 0.16 f	0.39 ± 0.019 e	1.14
	LP	F	0.94 ± 0.048 a	4.05 ± 0.07 h	1.86 ± 0.64 g	0.26 ± 0.013 f	0.89
	LP	S	0.87 ± 0.058 f	6.97 ± 0.04 d	1.08 ± 0.53 f	0.36 ± 0.018 e	1.45
GDI	C	F	1.26 ± 0.066 h	4.89 ± 0.12 g	6.68 ± 0.75 h	0.76 ± 0.038 g	1.00
	C	S	1.23 ± 0.061 i	4.68 ± 0.11 g	8.02 ± 0.78 i	0.83 ± 0.040 h	1.05
	LP	F	1.06 ± 0.040 d	6.09 ± 0.18 c	7.18 ± 0.56 j	0.74 ± 0.037 g	1.04
	LP	S	1.10 ± 0.21 d	5.36 ± 0.47 a	8.12 ± 0.59 i	0.86 ± 0.043 i	1.10

Means (± SD) in columns followed by different letters are significantly different (*n* = 9; *p* ≤ 0.05). C—control sprouts, LP—sprouts enriched with the probiotic, BE—buffer extract, GD—extract after gastric digestion, GDI—extract after gastrointestinal digestion. ABTS—ability to quench ABTS radicals; RP—reducing power; CHP—chelating power; OH—ability to quench OH radicals; TAI—total antioxidant index; TE—Trolox equivalents, EDTA—EDTA equivalents.

## Data Availability

The data presented in this study are available on request from the corresponding author.

## Sample Availability

Samples of the compounds are not available from the authors.
